# Retraction: Effect of intraoperative ventilation strategies on postoperative pulmonary complications: a meta-analysis

**DOI:** 10.3389/fsurg.2023.1220302

**Published:** 2023-06-06

**Authors:** Frontiers Editorial Office

A Retraction of the Systematic Review Article Effect of intraoperative ventilation strategies on postoperative pulmonary complications: a meta-analysis By Lei M, Bao Q, Luo H, Huang P and Xie J. (2021) Front. Surg. 8:728056. doi: 10.3389/fsurg.2021.728056

The journal and Chief Editors retract the 4 October 2021 article cited above.

Following publication, concerns were raised regarding abnormal similarities with the contents of other articles published by unrelated research groups. A subsequent investigation, which was conducted in accordance with Frontiers' policies, raised strong concerns over the authorship of the articles, resulting in a loss of confidence in the findings presented in the article.

The authors have not responded to this retraction.

This retraction was approved by the Chief Editors of Frontiers in Surgery and the Chief Executive Editor of Frontiers.

